# 

**Published:** 1860-05

**Authors:** 


### LIST OF COLLABORATORS.

ARMOR, S. G., M.D.,
Late Professor of Pathology and Clinical Medicine, Missouri Medical College.

ATKINSON, WM. B., M.D.,
Lecturer on Obstetrics, and Diseases of Women and Children, Philadelphia.

ATLEE, JOHN L., M.D.,
Late President Pennsylvania State Medical Society.

ATLEE, WALTER F., M.D.,
Philadelphia.

BARCLAY, ROBERT G., M.D.,
Jerusalem, Syria.

BELL, JOHN, M D.,
Philadelphia, formerly Professor of Medicine, Medical College of Ohio.

BEMISS, S. M., M.D.,
Professor of Clinical Medicine, University of Louisville.

BLACKBURN, L. P., M.D.,
Natchez, Miss.

BLACKIE, GEORGE C., M.D.,
Professor of Botany, University of Nashville.

BLACKMAN, G. C., M.D.,
Professor of Surgery, Medical College of Ohio.

BOBBS, J. L., M.D.,
Formerly Professor of Anatomy, Indianapolis.

BOWEN, W. S., M.D.,
New York.

BOZEMAN, N., M.D.,
Montgomery, Ala.

BRADFORD, J. T., M.D.,
Augusta, Ky.

BRINTON, JOHN H., M.D.,
Lecturer on Operative Surgery, Philadelphia.

BUMSTEAD, FREEMAN J., M.D.,
New York.

CARNOCHAN, J. M., M.D.,
Professor of Surgery in the New York Medical College.

CHIPLEY, W. S., M.D.,
Professor of Medicine, Transylvania University.

CLAPP, A., M.D.,
New Albany, Indiana.

CLIFFE, D. B., M.D.,
Franklin, Tenn.

DA COSTA, J., M.D.,
Lecturer on the Practice of Medicine at the Philadelphia Medical Association.

DICKSON, SAMUEL H., M.D.,
Professor of Medicine, Jefferson Medical College.

DUNGLISON, RICHARD J., M.D.,
Philadelphia.

DUPIERRIS, M., M.D.,
Havana, Cuba.

EDGAR, W. F., M.D.,
United States Army.

FARRAR, S. C., M.D.,
Jackson, Miss.

FENNER, C. S., M.D.,
Memphis, Tenn.

FISCHER, EMIL, M.D.,
Philadelphia.

FLINT, AUSTIN, M.D.,
Professor of Clinical Medicine, Auscultation, and Percussion, New Orleans School of Medicine.

FRICK, CHARLES, M.D.,
Professor of Materia Medica, University of Maryland.

GADBURY, W. Y., M.D.,
Benton, Miss.

GIBBS, O. C., M.D.,
Chautauque County, New York.

HALL, J. P., M.D.,
Glasgow, Ky.

HAMMOND, WM. A., M.D.,
United States Army.

HARDIN, JOHN, M.D.,
Professor of Obstetrics, Kentucky School of Medicine, Louisville.

HARTSHORNE, EDWARD, M.D.,
Surgeon to the Pennsylvania Hospital.

HASKINS, E. B., M.D.,
Professor of Medicine, Shelby Medical College, Nashville, Tenn.

HENTZ, C. A., M.D.,
Marianna, Florida.

HEWSON, ADDINELL, M.D.,
One of the Surgeons to Wills Hospital for Diseases of the Eye, Philadelphia.

HOLLINGSWORTH, SAMUEL L., M.D.,
Formerly Editor of the Medical Examiner, Philadelphia.

JEWELL, WILSON, M.D.,
Philadelphia, formerly President of the Quarantine and Sanitary Convention.

KEATING, WM. V., M.D.,
Lecturer on Obstetrics and the Diseases of Women, at the Phila. Medical Association.

KERR, JOHN G., M.D.,
Canton, China.

KUNKLER, G. A., M.D.,
Madison, Ia.

LOGAN, BEN., M.D.,
Shreveport, La.

LOPEZ, A., M.D.,
Mobile, Ala., formerly President of the Alabama State Medical Society.

MEIGS, J. AITKEN, M.D.,
Professor of the Institutes of Medicine in the Medical Department of Penna. College.

MITCHELL, S. WEIR, M.D.,
Lecturer on Physiology at the Philadelphia Medical Association.

MOREHOUSE, GEORGE R., M.D.,
Philadelphia.

RAPHAEL, B. I., M.D.,
New York.

REEVE, J. C., M.D.,
Dayton, Ohio,

REEVES, JAMES E., M.D.,
Philippi, Virginia.

RIVES, L. C., M.D.,
Formerly Professor of Midwifery, Medical College of Ohio.

SMITH, J. LAWRENCE, M.D.,
Professor of Chemistry, University of Louisville.

SNEED, W. C., M.D.,
Frankfort, Ky., formerly President of Kentucky State Medical Society.

SPILMAN, C. H., M.D.,
Harrodsburg, Ky., formerly President of Kentucky State Medical Society.

STORER, HORATIO R., M.D.,
Formerly one of the Physicians to the Boston Lying-in Hospital, Boston, Mass.

SUTTON, GEO., M.D.,
Aurora, Ia.

SUTTON, W. L., M.D.,
Georgetown, Ky., formerly President Kentucky State Medical Society.

THOMPSON, S., M.D.,
Albion, Ill., formerly President of the Illinois State Medical Society.

TODD, L. BEECHER, M.D.,
Lexington, Kentucky.

WILLIAMS, E., M.D.,
Cincinnati, Ohio.

### Bi-Monthly Bibliographical Record.

AMERICAN.

Nature in Disease; Illustrated in Various Discourses and Essays; to which are added Miscellaneous Writings, chiefly on Medical Subjects. By Jacob Bigelow, M.D., etc. 12mo., pp. 410. Second edition. New York: S. S. & W. Wood, 1860. (From the publishers.)

Brief Expositions of Rational Medicine; to which is prefixed the Paradise of Doctors, a Fable. By Jacob Bigelow, M.D., etc. Second edition, 8vo., pp. 69. New York: S. S. & W. Wood, 1860. (From the publishers.)

An Epitome of Braithwaite's Retrospect of Practical Medicine and Surgery. By Walter S. Wells, M.D. 8vo. Parts ii. and iii., pp. 305–912. New York, 1860. (From the author.)

Annual Address of the Events of the Year, delivered before the Philadelphia County Medical Society. By R. H. Coates, M.D., the retiring President. 8vo., pp. 28. Philadelphia, 1860. (From the author.)

Report of the Board of Managers of the Western Lunatic Asylum of the State of Kentucky, for the Years 1858–59. 8vo., pp. 63. (From the Secretary.)

Report of the Board of Managers and Superintendent of the Kentucky Eastern Lunatic Asylum, for the Years 1858–59. 8vo., pp. 39. (From Dr. Chipley.)

Proceedings of the Academy of Natural Sciences of Philadelphia. Pamphlet. 8vo., pp. 48. Philadelphia, 1860. (From the Secretary.)

Seventeenth Annual Report of the Managers of the State Lunatic Asylum of New York, for 1859. 8vo., pp. 36. (From Dr. Gray.)

Successful Treatment of a Case of Ligamentous Union of Fractured Radius and Ulna, by Drilling and Wiring, after Failure of other Means. By E. K. Sanborn, M.D., etc. Reprint. pp. 6. (From the author.)

Report of the Board of Managers of the Hospital of the Protestant Episcopal Church in Philadelphia. 8vo., pp. 32. Philadelphia, 1860. (From the Secretary.)

FOREIGN.

De l'Hématocèle Rétro-Utérine et des Épanchements Sanguins non Enkystés de la Cavité Peritoneale du Petit Bassin, Consideres comme Accidents da la Menstruation. Par Auguste Voisin. 8vo., pp. 359. Paris: J. B. Balliere et Fils, 1860. (From the publishers.)

On the Coagulation of the Blood in the Venous System during Life. By George Murray Humphry, M.D., etc. Reprint. 8vo., pp. 42. London: Macmillan & Co., 1859. (From the author.)

Dental Anomalies and their Influence upon the Production of the Diseases of the Maxillary Bones. By Am. Forget, M.D., etc. Translated from the French. 8vo., pp. 34. Philadelphia: Jones & White, 1860. (From the publishers.)

On the Causation and Prevention of Disease. By John Parkin, M.D., etc. 8vo., pp. 191. London: Churchill, 1859. (From the author.)

The Anatomy of the Human Lung. An Essay for which was awarded the Fothergillian Gold Medal of the Medical Society of London. By A. T. Houghton Waters, M.R.C.P., etc. 8vo., pp. 233. London: Churchill, 1860. (From the author.)

On the Intimate Structure, and the Distribution of the Blood-vessels of the Human Lung. By A. T. H. Waters, M.R.C.P., etc. Reprint. pp. 12. London, 1859. (From the author.)

On the Diseases of the Ear; their Nature, Diagnosis, and Treatment. By Joseph Toynbee, F.R.S., etc. 8vo., pp. 440. Philadelphia: Blanchard & Lea, 1860. (From the publishers.)

Cases of Amputations with Acupressure. By J. Y. Simpson, M.D., etc. Reprint. pp. 8. Edinburgh, 1860. (From the author.)

Traité d'Anatomie Chirurgicale, et de Chirurgie Experimentale. Par J. F. Malgaigne. Second edition. 2 vols. 8vo., pp. 784–880. Paris: J. B. Balliere et Fils, 1859.

Compendium der Physiologie des Menschen, mit Einschluss der Entwickelsengsgeschichte. Von A. Fick. 8vo. Vienna: Braumüller, 1859.

Traité Pratique des Maladies Nerveuses. Par Dr. Bourguignon, etc. Vol. i., 8vo., pp. 656. Paris: Germer Balliere, 1860.

A New and Rational Explanation of the Diseases peculiar to Infants and Mothers; with Obvious Suggestions for their Prevention and Cure. By Thomas Ballard, M.R.C.S., etc. Post 8vo. London: Churchill, 1860.

Imperfect Digestion; its Causes and Treatment. By Arthur Leared, M.B., etc. Fcap. 8vo. London: Churchill, 1860.

